# Kif21a deficiency leads to impaired glomerular filtration barrier function

**DOI:** 10.1038/s41598-023-46270-1

**Published:** 2023-11-06

**Authors:** Hanna Riedmann, Séverine Kayser, Martin Helmstädter, Daniel Epting, Carsten Bergmann

**Affiliations:** 1 Department of Medicine IV, Faculty of Medicine, Medical Center-University of Freiburg, Breisacher Str.113, 79106 Freiburg, Germany; 2 Limbach Genetics, Medizinische Genetik Mainz, Haifa-Allee 38, 55128 Mainz, Germany

**Keywords:** Cell biology, Developmental biology, Genetics, Molecular biology, Diseases, Medical research, Molecular medicine

## Abstract

The renal glomerulus represents the major filtration body of the vertebrate nephron and is responsible for urine production and a number of other functions such as metabolic waste elimination and the regulation of water, electrolyte and acid–base balance. Podocytes are highly specialized epithelial cells that form a crucial part of the glomerular filtration barrier (GFB) by establishing a slit diaphragm for semipermeable plasma ultrafiltration. Defects of the GFB lead to proteinuria and impaired kidney function often resulting in end-stage renal failure. Although significant knowledge has been acquired in recent years, many aspects in podocyte biology are still incompletely understood. By using zebrafish as a vertebrate in vivo model, we report a novel role of the Kinesin-like motor protein Kif21a in glomerular filtration. Our studies demonstrate specific Kif21a localization to the podocytes. Its deficiency resulted in altered podocyte morphology leading to podocyte foot process effacement and altered slit diaphragm formation. Finally, we proved considerable functional consequences of Kif21a deficiency by demonstrating a leaky GFB resulting in severe proteinuria. Conclusively, our data identified a novel role of Kif21a for proper GFB function and adds another piece to the understanding of podocyte architecture and regulation.

## Introduction

Human chronic kidney diseases (CKD) are a major global health burden that affect more than 10% of the global population^1^. Genetic and environmental factors influencing the function of the glomerular filtration barrier (GFB) are the leading cause of most CKDs^2–4^. Clinically, GFB disease is classified as steroid-sensitive or steroid-resistant nephrotic syndrome (SSNS or SRNS, respectively), minimal change disease (MCD) and focal segmental glomerulosclerosis (FSGS). The GFB represents a highly specialized three-layered capillary wall comprising fenestrated endothelial cells, glomerular basement membrane (GBM) and podocytes. While injuries of the glomerular endothelium and defects of the GBM can cause pathological conditions such as albuminuria, the majority of reported glomerular diseases highlight the critical role of podocytes in GFB function^5,6^. Podocytes are post-mitotic highly differentiated epithelial cells that cover the outside of the GBM and closely envelope the glomerular capillaries. Recent advances in microscopy technologies allowed high-resolution visualization of the GFB ultrastructure, and revealed that podocytes form extensions (foot processes) interdigitating with those of neighboring podocytes thereby forming membrane-like slits, commonly known as slit diaphragms, and responsible for ultrafiltration^7–9^. *NPHS1* and *NPHS2* encode for Nephrin and Podocin, respectively, and resemble the main constituents of the slit diaphragm that cause nephrotic syndrome when mutated^3^. Recently, pathogenic variants in Kidney Ankyrin Repeat-containing Protein 1 (KANK1), KANK2 and KANK4 have been identified in individuals affected with nephrotic syndrome^10^. KANKs have a unique structure containing coiled-coil motifs and ankyrin-repeats in their N- and C-terminal regions, respectively, and a Kank N-terminal (KN) motif^11^. Localization studies revealed specific localization of KANK proteins at the glomerular podocytes. In the same report, knockdown studies using in vivo model organisms *Drosophila melanogaster* and zebrafish demonstrated a critical role of KANKs in GFB function^10^. KANKs act within a cortical microtubule stabilization complex (including LL5b, Liprin-α1, Liprin-β1 and KIF21A) and are thereby involved in the control of cytoskeleton formation by regulating actin polymerization^12^. KANKs functionally interact with Kinesin-4 family member KIF21A thereby recruiting KIF21A to the cell cortex^13,14^. KIF21A consists of an N-terminal motor domain, a coiled-coil domain in the middle part and a C-terminal WD40 domain^15^. An interaction of the motor domain with the third coiled-coil domain results in KIF21A autoinhibition^16^. Structural, biochemical and cellular analyses revealed that a specific ankyrin-repeat binding domain ANKRD of KANKs interacts with a short binding domain (KANK binding domain, KBD) located in the middle part of KIF21A^17–20^. Functional analyses showed that KIF21A acts as an important microtubule growth inhibitor at the cell cortex^14^. In addition, a recent report demonstrated an essential role for *Caenorhabditis elegans* KIF21A orthologue KLP-12 in axonal length control by inhibiting microtubule dynamics^21^. Gain-of-function variants in *KIF21A* are associated with congenital fibrosis of the extraocular muscles type 1 (CFEOM1) which is an autosomal dominant neurodevelopmental disorder affecting the oculomotor nerve^22–24^. Clinical features of CFEOM1 include strabismus, ptosis, blepharoptosis, ophthalmoplegia and amblyopia^25^. In vivo studies using either *KIF21A* knock-in mice harboring the most common CFEOM1 genetic variant R954W or *KIF21A* knock-out mice recapitulated human CFEOM or revealed death within the first 24 h and no CFEOM-associated features, respectively^16^. Most recently, a single homozygous loss-of-function variant in *KIF21A* has been identified in individuals affected by severe neurogenic fetal akinesia (FA) sequence with arthrogryposis of multiple joints, pulmonary hypoplasia and facial dysmorphism^26^. The KIF21A interaction with KANKs strongly supports the hypothesis that KIF21A might have much more diverse functions in the organism than currently known such as a role in podocyte and GFB function. Notably, KIF21A protein was identified in comprehensive proteome analyses of cultured human podocytes and of native mouse podocytes^27,28^. In addition, a physical and functional KIF21A-KANK2 interaction has been demonstrated in cultured human podocytes^20^. We therefore analysed a potential GFB function of Kif21a using the zebrafish as a well-established vertebrate model organism to study kidney development and function. We report here specific localization of Kif21a in the glomerulus and that Kif21a deficiency leads to functional defects in podocyte morphology and a leaky GFB resulting in proteinuria. Taken together, our data identified Kif21a as a novel nephrotic syndrome associated candidate playing a major role in proper GFB function in zebrafish.

## Results

### Expression of *kif21a*/Kif21a during zebrafish development

**Figure 1:**
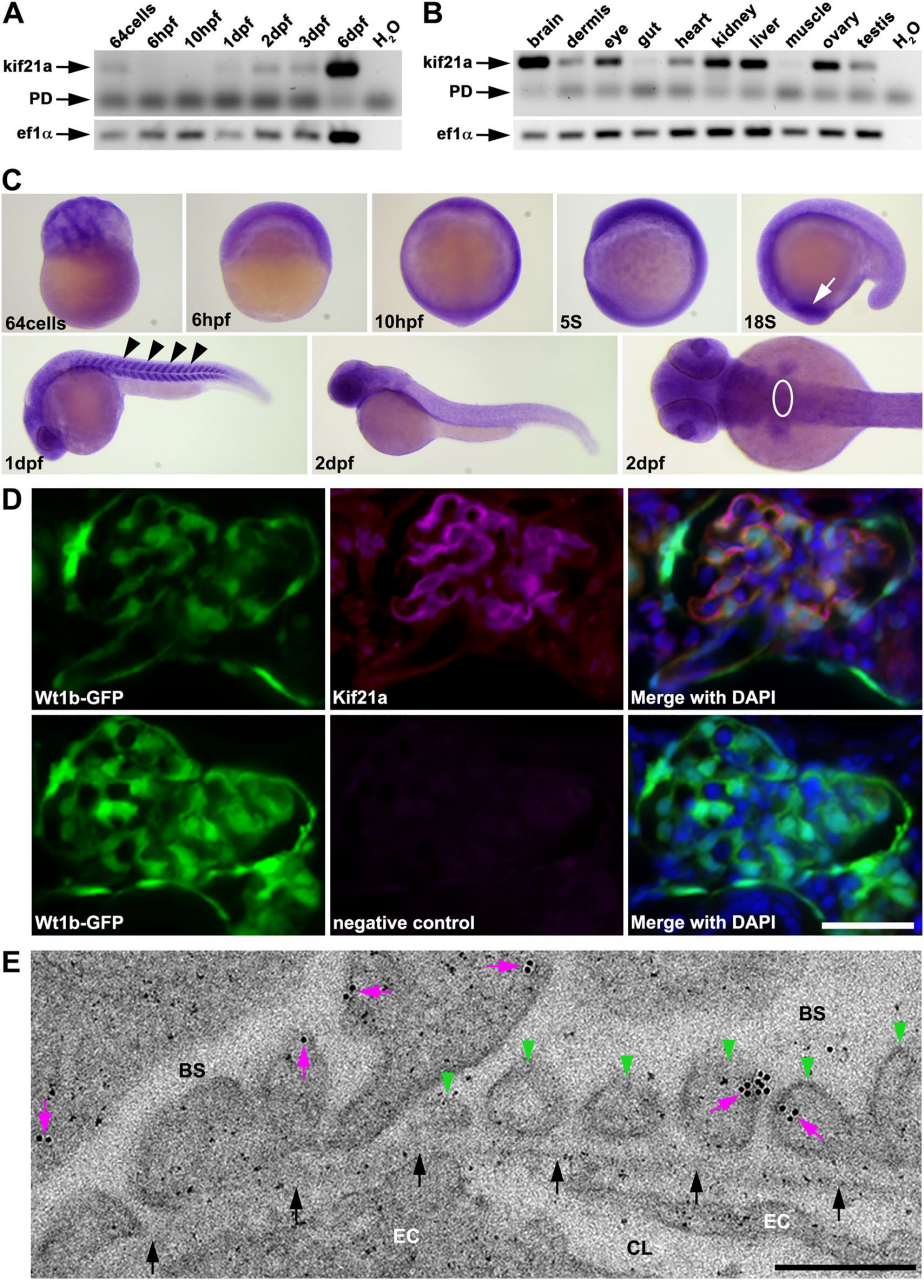
Kif21a shows a specific expression during zebrafish development and localized to the glomerular filtration barrier. (**A, B**) Expression analysis of *kif21a* in zebrafish using semiquantitative reverse-transcription polymerase chain reaction (RT-PCR) on cDNA of different developmental embryonic stages (**A**) and adult organs (**B**). H_2_O served as negative control and *ef1α* as loading control; Primer dimer (PD). The grouping of gel images cropped from different parts of the same gel image is delineated with white spaces. (**C**) Whole-mount in situ hybridization (WISH) analysis detecting temporal and spatial *kif21a* expression at different stages during zebrafish embryonic development (white arrow marks the optic stalk at 18 somite stage; black arrowheads mark the somites at 1 day post-fertilization (dpf); white circle depicts the glomerular area). (**D**) Glomerular sections of 5dpf old *Tg(wt1b:GFP)* zebrafish embryos (green) that were immunostained for Kif21a (magenta) and counterstained with DAPI (blue) as a nuclear marker. A respective immunostaining without the secondary antibody served as negative control. Scale bar represents 25 µm. (**E**) Representative electron micrograph showing immunogold-labelling of Kif21a (magenta arrows) in the glomerular region from 5dpf old zebrafish embryos. Green arrowheads point to podocyte foot processes and black arrows mark the glomerular basement membrane. Bowman’s space (BS), capillary lumen (CL), fenestrated endothelial cell (EC). Scale bar represents 250 nm. Unprocessed gel images for (**A** and **B**) are presented in Supplementary Fig. S2.

To analyse the temporal and spatial expression of *kif21a* in zebrafish, we performed semiquantitative RT-PCR on cDNA of different stages of embryonic development and of adult organs (Fig. 1A, B). These analyses revealed maternal expression of *kif21a*, and whereas *kif21a* expression is not detectable at 6 h post-fertilization (hpf) and 10hpf, it was detected from days 1 to 6 post-fertilization (dpf) (Fig. 1A). In adult zebrafish, we detected only minor *kif21a* expression in the dermis, gut, heart, muscle and testis, whereas *kif21a* is highly expressed in brain, eye, kidney, liver and ovary (Fig. 1B). Whole-mount in situ hybridization (WISH) studies confirmed the maternal expression of *kif21a* at the stage of 64cells and revealed ubiquitous expression at all stages analysed. There was specific expression in the optic stalk and somites at 18 somite stage and 1dpf, respectively, but we could not detect any specific *kif21a* expression in the glomerular region at 2dpf using WISH (Fig. 1C). We performed co-immunostaining studies for Kif21a on cryosections of 5dpf old *Tg(wt1b:GFP)* zebrafish embryos displaying glomerular GFP expression. Subsequent analyses revealed partial co-labelling of Kif21a and GFP, clearly demonstrating Kif21a expression in the glomerulus (Fig. 1D). In addition, immunogold electron microscopy (EM) revealed membrane associated localization of Kif21a predominantly at the lateral side of podocyte foot processes (Fig. 1E).

### Knockdown of Kif21a leads to podocyte defects resulting in proteinuria in zebrafish

**Figure 2:**
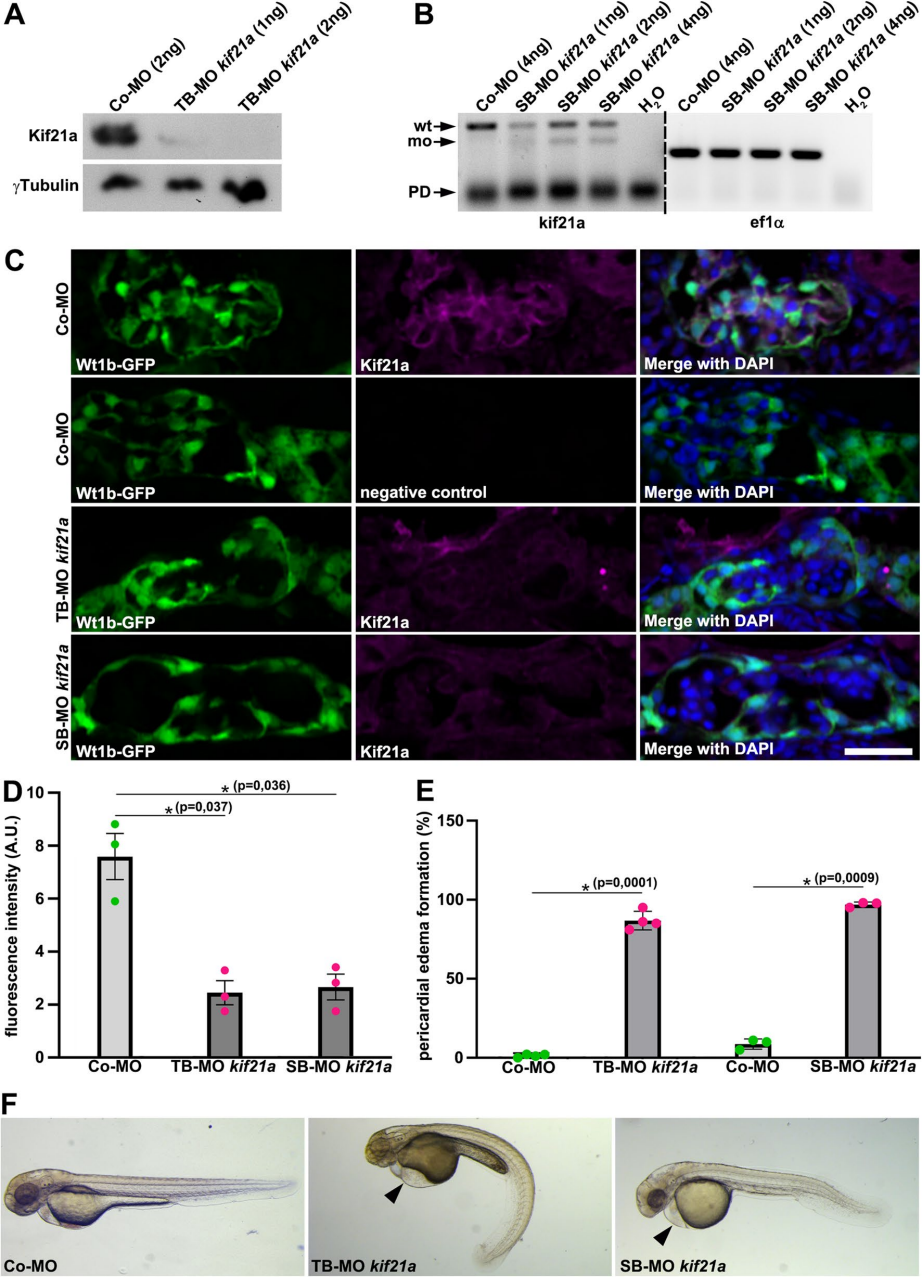
Kif21a deficiency results in pericardial edema formation and reduced glomerular Kif21a signal in zebrafish. (**A**) Immunoblotting studies on zebrafish lysates of 4dpf old embryos injected with Control (Co)-MO (2 ng) or translation-blocking Morpholino (TB-MO) *kif21a* (1 or 2 ng) using a specific Kif21a antibody.* γ*Tubulin served as loading control. The grouping of blot images cropped from different parts of the same blot image is delineated with a white space. (**B**) Expression analysis of *kif21a* using semiquantitative RT-PCR on cDNA of Co-MO (4 ng) or splice-blocking Morpholino (SB-MO) *kif21a* (1, 2 or 4 ng). Black arrows point to kif21a wildtype (wt) PCR product and morphant (mo) PCR splice product; Primer dimer (PD). H_2_O served as negative control and *ef1α* as loading control; dividing lines indicate different contrast from different parts of the same gel image. (**C**) Glomerular sections of 5dpf old *Tg(wt1b:GFP)* zebrafish embryos (green) that were injected with Co-MO (6 ng), TB-MO *kif21a* (2 ng) or SB-MO *kif21a* (6 ng), immunostained for Kif21a (magenta) and counterstained with DAPI (blue) as a nuclear marker. A respective immunostaining without the secondary antibody of a Co-MO injected embryo served as negative control. Scale bar represents 25 µm. (**D**) Quantification reveals significant reduced Kif21a levels in the glomerulus of embryos injected with TB-MO *kif21a* (2 ng) or SB-MO *kif21a* (6 ng) compared to Co-MO (6 ng) injected embryos; Arbitrary Unit (A.U.). The barchart represents the pooled data from 3 different embryos for each condition. (**E**) Quantification of pericardial edema formation of 2dpf old zebrafish embryos injected with Co-MO (6 ng), TB-MO *kif21a* (2 ng) or SB-MO *kif21a* (6 ng). The barchart represents the pooled data from 3 or 4 independent experiments for each condition, respectively; number (n) of total embryos analysed: Co-MO, n = 390; TB-MO *kif21a*, n = 384 and Co-MO, n = 290; SB-MO *kif21a*, n = 290. (**F**) Bright-field images of zebrafish embryos at 2dpf injected with Co-MO (6 ng), TB-MO *kif21a* (2 ng) or SB-MO *kif21a* (6 ng) (black arrowhead points to pericardial edema formation). Unprocessed blot and gel images (**A** and **B**, respectively) are presented in Supplementary Fig. S3.

**Figure 3:**
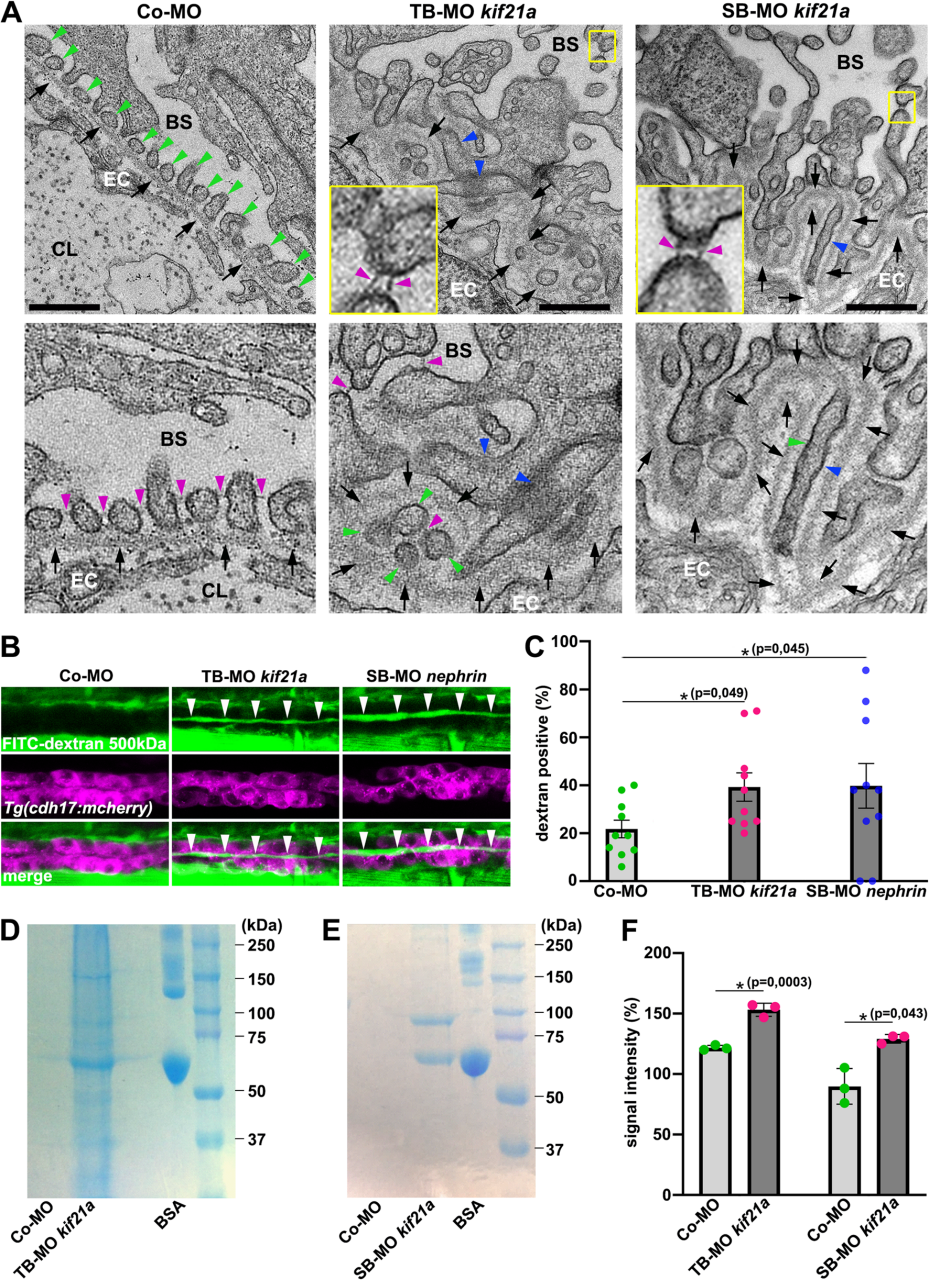
Kif21a deficiency results in defective podocyte morphology leading in a leaky glomerular filtration barrier and proteinuria. (**A**) Representative electron micrographs of glomerular region from 5dpf old zebrafish embryos injected with Co-MO (6 ng), TB-MO *kif21a* (1 ng) or SB-MO *kif21a* (6 ng). Green, blue and magenta arrowheads point to podocyte foot processes, podocyte foot process effacements and slit diaphragms, respectively. Black arrows point to glomerular basement membrane. Yellow-framed boxes represent magnifications of respective insets. Bowman’s space (BS), capillary lumen (CL), fenestrated endothelial cell (EC). Scale bars represent 500 nm. (**B**) Representative confocal images of 4dpf old *Tg(cdh17:mcherry)* embryos (expressing red fluorescent reporter protein in the pronephric tubules) injected with Co-MO (2 ng), TB-MO *kif21a* (1 ng) or SB-MO *nephrin* (2 ng) that were injected with 500 kDa FITC-dextran into the common cardinal vein at 80hpf. White arrowheads indicate the presence of 500 kDa FITC-dextran in the pronephric tubule lumen. (**C**) Quantification of 4dpf old embryos injected with Co-MO (2 ng), TB-MO *kif21a* (1 ng) or SB-MO *nephrin* (2 ng) that show the presence of 500 kDa FITC-dextran in their pronephric tubule lumen. The knockdown of *nephrin*, a well-established zebrafish model causing dysfunction of the GFB and proteinuria, served as positive control. The barchart represents the pooled data from 10 independent experiments for each condition; number (n) of total embryos analysed: Co-MO, n = 98; TB-MO *kif21a*, n = 100; SB-MO *nephrin*, n = 92). (**D**) SDS-PAGE analysis of processed urine samples of embryos injected with Co-MO (2 ng) or TB-MO *kif21a* (2 ng) for the identification of proteinuria. Bovine serum albumin (BSA) was used as positive control. (**E**) SDS-PAGE analysis of processed urine samples of embryos injected with Co-MO (6 ng) or SB-MO *kif21a* (6 ng) for the identification of proteinuria. BSA was used as positive control. (**F**) Quantification of SDS-PAGE signal intensity of embryos injected with Co-MO (2 or 6 ng), TB-MO *kif21a* (2 ng) or SB-MO *kif21a* (6 ng). The barchart represents the pooled data from 3 independent experiments for each condition. Unprocessed SDS-PAGE images (**D** and** E**) are presented in Supplementary Fig. S4.

We performed a Morpholino (MO)-based knockdown approach to analyse Kif21a function in zebrafish (Fig. 2). Knockdown with either a translation- or splice-blocking MO targeting *kif21a* (TB-MO *kif21a* or SB-MO *kif21a*) resulted in significant reduced Kif21a protein levels or splice modification of *kif21a*, respectively (Fig. 2A, B). In addition, immunohistochemistry for Kif21a on *Tg(wt1b:GFP)* embryos injected with either TB- or SB-MO *kif21a*, revealed a strong reduction of Kif21a signal intensity compared to the control, confirming MO specificity (Fig. 2C, D). Kif21a deficient embryos displayed pericardial edema formation that appeared at 2dpf and aggravated until 5dpf, and frequently extended into the intravascular compartment of the yolk sac (Fig. 2E, F). Notably, this phenotype resembles a well-described indication for defective GFB function in zebrafish as it has been reported in embryos that are deficient for important slit diaphragm components such as Nephrin and Podocin^29–31^. EM analyses on glomerular sections of zebrafish embryos deficient for Kif21a or Podocin revealed defective podocyte formation, known as podocyte foot process effacement. In addition, Kif21a morphants displayed an overall disorganized localization pattern of the podocyte foot processes, i.e. podocyte foot processes are frequently not localized at the GBM while still forming slit diaphragms with foot processes of adjacent podocytes in the Bowman´s capsule, and we also detected podocyte foot processes disturbing the GBM architecture (Fig. 3A and Suppl. Fig. S1). Next, we performed a well-accepted glomerular filtration permeability assay in zebrafish by analysing the clearance of 500 kDa fluorescein isothiocyanate (FITC) conjugated dextran in Kif21a morphant *Tg(cdh17:mcherry)* embryos compared to respective controls (Fig. 3B, C)^30,32,33^. These analyses revealed statistically significant clearance of 500 kDa FITC-dextran in embryos deficient for either Kif21a or Nephrin compared to the control (Fig. 3C). Being aware that this in vivo proteinuria assay has some known disadvantages (e.g. requirement of unphysiological macromolecule FITC-dextran intravenous injection, linear shaped 500 kDa dextran might pass the filtration barrier), we performed another established permeability assay that analyses the excretion of proteins in urine^34^. For this purpose, we collected and concentrated the water from small petri dishes, containing either control or Kif21a deficient embryos, and subsequently analysed for protein content using SDS-PAGE. By these results, we could clearly determine proteinuria in Kif21a deficient embryos (Fig. 3D–F). Taken together, our results obtained from different glomerular filtration permeability assays demonstrate a defective GFB function in Kif21a morphant embryos.

## Discussion

Loss of podocyte integrity due to cytoskeleton dysregulation plays a major role in proteinuric chronic kidney disease (CKD)^35^. Hence, proper temporal and spatial control of cytoskeletal dynamics of the podocyte and its adjacent structures is crucial for decent glomerular filtration and a better understanding key for the design of clinical studies and therapeutic approaches. While intermediate filaments and microtubules predominantly shape the primary processes of podocytes, any secondary processes are mainly based on actin cytoskeletal dynamics^36^. KANK proteins in which pathogenic alterations lead to proteinuria and nephrotic syndrome in affected individuals have been previously functionally demonstrated to be indispensable for proper cytoskeletal formation in podocytes^10^. Notably, some functional interrelationships do exist for KANKs and the microtubule plus-end directed motor protein KIF21A. Both, KANK proteins and KIF21A, drive the stabilization of the decisive microtubule complex^12^. In line, KIF21A is recruited to the cell cortex via a direct interaction with KANKs^13,14^. In addition, KIF21A has been shown to be crucial for microtubule organization by inhibiting microtubule growth at the cell cortex^14,21^. Low levels of *KIF21A* mRNA expression have been detected in human fetal and adult kidney and KIF21A protein was identified in two independent proteome analyses of cultured human podocytes and native mouse podocytes, respectively^23,27,28^. Noteworthy, a physical and functional interaction of KIF21A with KANK2 has been already demonstrated in cultured human podocytes^20^.

In this study, we demonstrate a novel role of Kif21a in the establishment of a functional glomerular filtration barrier (GFB) in zebrafish, a suitable vertebrate in vivo model that is well-accepted for the analysis of human kidney disease such as proteinuric disorders. Zebrafish Kif21a (XP_021326429.1) has 68% identity and 86% similarity to its human orthologue (NP_001166935.1). Our studies revealed specific localization of Kif21a in the glomerulus that is comparable to the localization of other members of the apical microtubule stabilization complex^10^. Our glomerular filtration analyses demonstrated a leaky GFB in Kif21a deficient zebrafish embryos. Subsequent electron microscopy analyses revealed a severe glomerular phenotype displaying disorganized podocyte foot process formation. As Kif21a acts as a microtubule growth inhibitor, a reasonable explanation for this phenotype is uncontrolled microtubule growth leading to defects in the formation of podocyte foot processes and slit diaphragms that subsequently results in defective GFB function^14,21^. Conclusively, our data demonstrate a novel role of Kif21a in the regulation of podocyte foot process formation by which proper glomerular filtration is established and maintained in zebrafish. Although the zebrafish pronephros is much simpler in architecture compared to the mammalian nephron, it is of particular interest to researchers. It consists of a pair of segmented pronephric tubules sharing a fused glomerulus and has striking structural and functional similarities to the mammalian adult nephron^37,38^. Hence, recent research used the zebrafish as vertebrate model organism to study human renal diseases including glomerular diseases^39–41^. However, additional analyses on KIF21A functions in mammalian podocytes will be necessary to confirm our findings in the mammalian kidney.

It is tempting to speculate that mono- and/or biallelic pathogenic variants in *KIF21A* may cause proteinuria or nephrotic syndrome in affected individuals. To date, genetic variants in *KIF21A* were identified only in patients affected with CFEOM1 or FA with arthrogryposis multiplex^22–24,26^. All described CFEOM1-causing variants are thought to render KIF21A in a constitutively active state, and are therefore considered to lead to gain-of-function (GOF) of the resulting protein. In contrast, individuals affected with a severe neurogenic FA sequence with arthrogryposis of multiple joints were recently identified to harbour biallelic loss-of-function (LOF) variants in *KIF21A*. In addition to the mutational type and functional character such as GOF and LOF and their underlying gene dosage, the resulting phenotype might also depend on the exact position of alterations within the protein and if one or both gene copies are affected. Pleiotropy has been described for various genes and it is well conceivable that variants in *KIF21A* may also cause a wider phenotypic spectrum than currently described. There are quite a number of genes for which clinical manifestations of the kidney, eye and/or the central/peripheral nervous system have been demonstrated when mutated^42–45^. Notably, podocytes share common characteristics with neurons, further emphasizing the close interrelationship and similarities between kidney and nervous system^36,46^. Mutational studies in respective patient cohorts will be needed to answer this question. Given the results presented in our study and those published in the literature, individuals with nephrotic syndrome may have pathogenic variants in *KIF21A*, encoding a direct interaction partner of KANK1 and KANK2.

## Materials and methods

### Zebrafish lines and embryo maintenance

The fish used in this study were maintained at the Zebrafish Facility of the Medical Center of the University of Freiburg. All animal work has been conducted according to relevant national and international guidelines^47^. The study was approved by the Institutional Animal Care of the Medical Center of the University of Freiburg and the Regional Council Freiburg (permit ID G-16/89). All methods were carried out in accordance with ARRIVE guidelines. Zebrafish were maintained and the embryos were staged as previously described^48^. The following strains were used: AB/TL wildtype (WT), *Tg(wt1b:EGFP)*^49^ and *Tg(cdh17:mcherry)*^32^.

### Reverse-transcription polymerase chain reaction (RT-PCR) analysis

Semiquantitative RT-PCR was performed to determine expression of zebrafish *kif21a* during embryonic development and in adult organs. Total RNA from entire zebrafish embryos or adult zebrafish organs was extracted with the RNeasy Kit (Qiagen), followed by complementary DNA (cDNA) synthesis with the ProtoScript First Strand cDNA Synthesis Kit (Promega). Analysis of zebrafish *ef1α* was used as a loading control. The following primers were used for PCR analysis: kif21a (forward: 5ʹ-ACACACAGCTGGAGAGAGAC-3ʹ, reverse: 5ʹ-GCTGCTCCTTCATCTGCTTC-3ʹ); ef1α (forward: 5ʹ-ATCTACAAATGCGGTGGAAT-3ʹ, reverse: 5ʹ-ATACCAGCCTCAAACTCACC-3ʹ).

### Antisense RNA synthesis and in situ analysis

Zebrafish *kif21a* was amplified from 5dpf zebrafish cDNA with primers (kif21a, forward: 5ʹ-TACCGTCTCCACCTCCTACA-3ʹ, reverse: 5ʹ-CTTGATGCCGTTGTCTCTGG-3ʹ), cloned into TOPO (Invitrogen) and linearized with corresponding restriction enzymes. Whole-mount in situ hybridization (WISH) analysis using Digoxigenin-labelled probes was performed as described^50^ using NBT (blue) (Roche) as substrate.

### Morpholino (MO) injection

Morpholino oligonucleotide (MO) injection was performed as described^51^. To attenuate possible off target effects, a p53 MO was co-injected 1.5-fold to the other MOs used^52^. The following translation/splice-blocking (TB/SB) antisense MOs (Gene Tools) were used for zebrafish:

TB-MO *kif21a* 5′-GGTGCTGTCGTCCATGATGTTAA-3′, SB-MO *kif21a* 5′-TCCAGCAGGTATTGAGACACAGACC-3′, SB-MO *podocin* 5'-TAGACTTACCTTCTCCAGGTCCCTC-3′^30^, SB-MO *nephrin* 5'-CGCTGTCCATTACCTTTCAGGCTCC-3′^30^, TB-MO *p53* 5'-GCGCCATTGCTTTGCAAGAATTG-3′^52^ and a Standard Control (Co)-MO.

### Immunoblotting

Immunoblotting (IB) and signal detection was performed as previously described^53^. The following antibodies were used for IB: anti-KIF21A (orb184767, Biorbyt, diluted 1:1000), anti‐*γ*Tubulin (clone GTU‐88, Sigma Aldrich, diluted 1:5000) and respective HRP‐conjugated antibodies (DAKO, diluted 1:5000).

### Cryosectioning

Embryos were kept until 5dpf in Danieau’s solution with PTU at 28.5 °C. They were sacrificed with tricaine and transferred into 4% paraformaldehyde (PFA) for incubation at 4 °C overnight. The embryos were rinsed 3 times for 5 min with 1xPBST and incubated in 30% sucrose overnight at 4 °C. The following day the embryos were embedded carefully in sagittal orientation in small plastic cases in TissueTek® O.C.T. that was polymerized over liquid nitrogen. Plastic cases were stored at −80 °C or used directly for sectioning.

### Immunohistochemistry

Cryosections were made on 5dpf embryos of *Tg(wt1b:GFP)* transgenic fish line with the aim of visualizing the glomeruli in this way for making orientation easier while sectioning. The GFP signal of the *Tg(wt1b:GFP)* fish could be observed in the area that comprised the pronephric glomeruli, the pronephric tubules and proximal parts of the pronephric tubules. 4 μm cryosections were made with a cryotome, and located by checking sections under a fluorescence microscope. Sections on glass slides were stored in ice-cold 1xPBS between the cutting and staining process. Before staining, the slides were dried carefully with a paper towel without damaging the sections, and then individual sections were circled with water-repellent slide marker pen to enable different AB-staining on one slide. For the following steps, slides were kept in a dark-wet-chamber. Sections were first blocked for 1 h at room temperature (RT) with 5% donkey serum and 5% BSA in 1xPBS. Blocking solution was replaced by anti-KIF21A (orb184767, Biorbyt) diluted in blocking solution (1:500) and incubated at 4 °C overnight. Nuc Blue Live Cell Stain (Hoechst 33342) was purchased from Thermo Fisher Scientific. As a control the incubation was only performed with the secondary antibody. After washing the sections 3 times for 10 min with 1xPBST, the secondary antibody (anti-rabbit, Cy3) 1:500 diluted in blocking solution was added and incubated for 1 h at RT. Again, 3 times washing with 1xPBST before mounting them with Diamond Mounting Medium (Invitrogen) and covering with coverslips. The slides were dried for 24 h in the dark at RT and subsequently they were stored at 4 °C until they were displayed under the microscope.

### Immunogold-labelling

On-grid immunogold-labelling was performed using anti-KIF21A (orb184767, Biorbyt, diluted 1:200) and goat anti-rabbit IgG 10 nm gold on gold Grids using Aurion solutions and protocol (Aurion, Wageningen, Netherlands). In brief, the grids were incubated in 0.05 M glycine in 1xPBS for 15 min to inactivate residual aldehydes and subsequently blocked with normal goat serum for 15 min and washed 2 times for 5 min in Aurion Incubation Solution. This was followed by incubation with primary antibody for 2 h at RT, washing steps (6 times for 5 min) and subsequent incubation with secondary antibody (diluted 1:20) for 2 h. The washed grids where then post fixed in 2% glutaraldehyde in 1xPBS and imaged.

### Fluorescent dye injection

Lysine fixable fluorescein isothiocyanate (FITC) conjugated dextran, 500 kDa (Molecular Probes; 25 mg/mL diluted 1:20 in H_2_O) was injected into the common cardinal vein of MO-injected 80hpf *Tg(cdh17:mcherry)* embryos, anesthetized with 0.4% tricaine in Danieau’s solution. Before injection of the fluorescent tracer sufficient blood circulation was checked by eye, judged by enough moving red blood cells. Afterwards the embryos were transferred to fresh Danieau’s solution with PTU for overnight incubation at 28.5 °C. At 96hpf embryos were checked again for proper blood circulation and subsequently analysed with a scanning confocal microscope for the presence of the 500 kDa dextran in the pronephric tubules.

### Proteinuria assay

The proteinuria assay was performed as previously described^34^. For each measurement, 100 MO-injected embryos were collected at 96 hpf, transferred to a small petri dish containing 5 ml fresh Danieau’s solution, and incubated at 28,5 °C for 24 h. At 120hpf and after ensuring all embryos in the petri dish were alive, 4 ml of Danieau’s solution was carefully collected and transferred into a 15 ml falcon tube. 1 ml ≥ 99% trichloroacetic acid (TCA, Sigma-Aldrich) was gently mixed with the 4 ml harvested Danieau’s solution and incubated for 1 h at 4 °C. Afterwards, the mixture was centrifuged at 13,000 rpm for 5 min at 4 °C. The supernatant was carefully removed, and the protein pellet was resuspended in 1 ml cold acetone and centrifuged at 13,000 rpm for 5 min at 4 °C. The washing with acetone was repeated, the pellet was air-dried for 10 min and then resuspended with 15 µL NuPage LDS Sample Buffer 4x (Thermo Fisher Scientific). After incubation at 70 °C for 10 min, the proteins were separated by SDS-PAGE, and the gel was stained with PageBlue Protein Staining Solution (Thermo Scientific) according to the manufacturer’s instructions.

### Microscopy and image acquisition

Confocal imaging of zebrafish embryos was performed using confocal microscope LSM510 ZEISS (ZEISS objectives: Achroplan NIR 40x/0.8 water-immersion). Zebrafish embryos were embedded in 1% low-temperature melting agarose (Biozym) in 30% Danieau’s solution. Vertical projections of recorded stacks were generated using LSM Examiner software (ZEISS). Bright-field images of whole-mount in situ embryo stains were taken using an Axioplan2 microscope with Axiocam camera and using Axiovision software (ZEISS). Immunostained cryosections of zebrafish embryos were analysed with an apotome microscope (ZEISS ApoTome). Embryos were analysed under a Leica MZ16 stereo-microscope (Leica, Solms, Germany), and non-confocal fluorescent images were obtained with a SPOT Insight Fire Wire System (Diagnostic Instruments, Sterling Heights, MI). For transmission electron microscopy (TEM), zebrafish embryos were fixed in EM fixative (4% PFA and 2% glutaraldehyde in 0.1 M sodium cacodylate buffer, pH 7.4) using a PELCO BioWave® Pro + laboratory microwave (Ted Pella Inc., Redding, CA, USA), followed by incubation overnight at 4 °C. In brief, fixed samples were washed 3 times in 0.1 M sodium cacodylate buffer before microwave assisted contrastation (1% OsO_4_ in H_2_O; 1% uranylacetat in H_2_O), dehydration (ethanol series), and embedding in Durcupan resin. Ultrathin sections (50–70 nm) were prepared using a Leica UC7 ultramicrotome (Leica microsystems, Vienna), mounted on grids and contrasted using lead citrate. Sections were imaged using a transmission electron microscope operated at 120 kV (Talos L120C TEM; Thermo Scientific, Eugene, OR, USA). All images were exported as TIFF files and imported into Adobe Photoshop software CS2 to arrange figures.

### Statistical analysis and quantification

Each figure shows the results of one experiment from a set of at least three independent experiments, and is typical of the set. Numbers of embryos used for analysis are indicated in the respective figure legend. Data were analysed by Student’s *t*-test (2-sided, unpaired); error bars represent the standard error of the mean (SEM). The presence of pericardial edema formation was quantified at 2dpf and monitored until 5dpf of zebrafish development. For the quantification of Kif21a fluorescence intensity, a region of interest (ROI), including the glomerular region, was selected. All intensity measurements were performed using ImageJ Fiji (https://fiji.sc/).

### Accession numbers

Corresponding GenBank accession number for zebrafish cDNA: *kif21a* (XM_021470754.1).

### Supplementary Information

Supplementary Information.

## Data Availability

The datasets used and/or analysed during the current study are available from the corresponding authors on reasonable request.
