# Supplementary material for: Kif21a deficiency leads to impaired glomerular filtration barrier function
Source: Sci Rep. 2023 Nov 6;13:19161. doi: 10.1038/s41598-023-46270-1 (PMC10628293; doi:10.1038/s41598-023-46270-1)
Supplement: Supplementary file 1 — Supplementary Information. [file 41598_2023_46270_MOESM1_ESM.docx]

**Supplementary Information**

**Kif21a deficiency leads to impaired glomerular filtration barrier function**

Hanna Riedmann^1^, Séverine Kayser^1^, Martin Helmstädter^1^, Daniel Epting^1,3,^* and Carsten Bergmann^1,2,3,^*

^1^ Department of Medicine IV, Faculty of Medicine, Medical Center-University of Freiburg, 79106 Freiburg, Germany.

^2^ Medizinische Genetik Mainz, Limbach Genetics, 55128 Mainz, Germany.

^3^ These authors contributed equally: Daniel Epting and Carsten Bergmann

* Corresponding authors

**Suppl. Fig. 1 Podocin deficiency results in defective podocyte morphology.**

**Suppl. Fig. 2 Respective unprocessed gel images.**

**Suppl. Fig. 3 Respective unprocessed blot and gel images.**

**Suppl. Fig. 4 Respective unprocessed SDS-PAGE images.**

**Suppl. Fig. 1 Podocin deficiency results in defective podocyte morphology.**


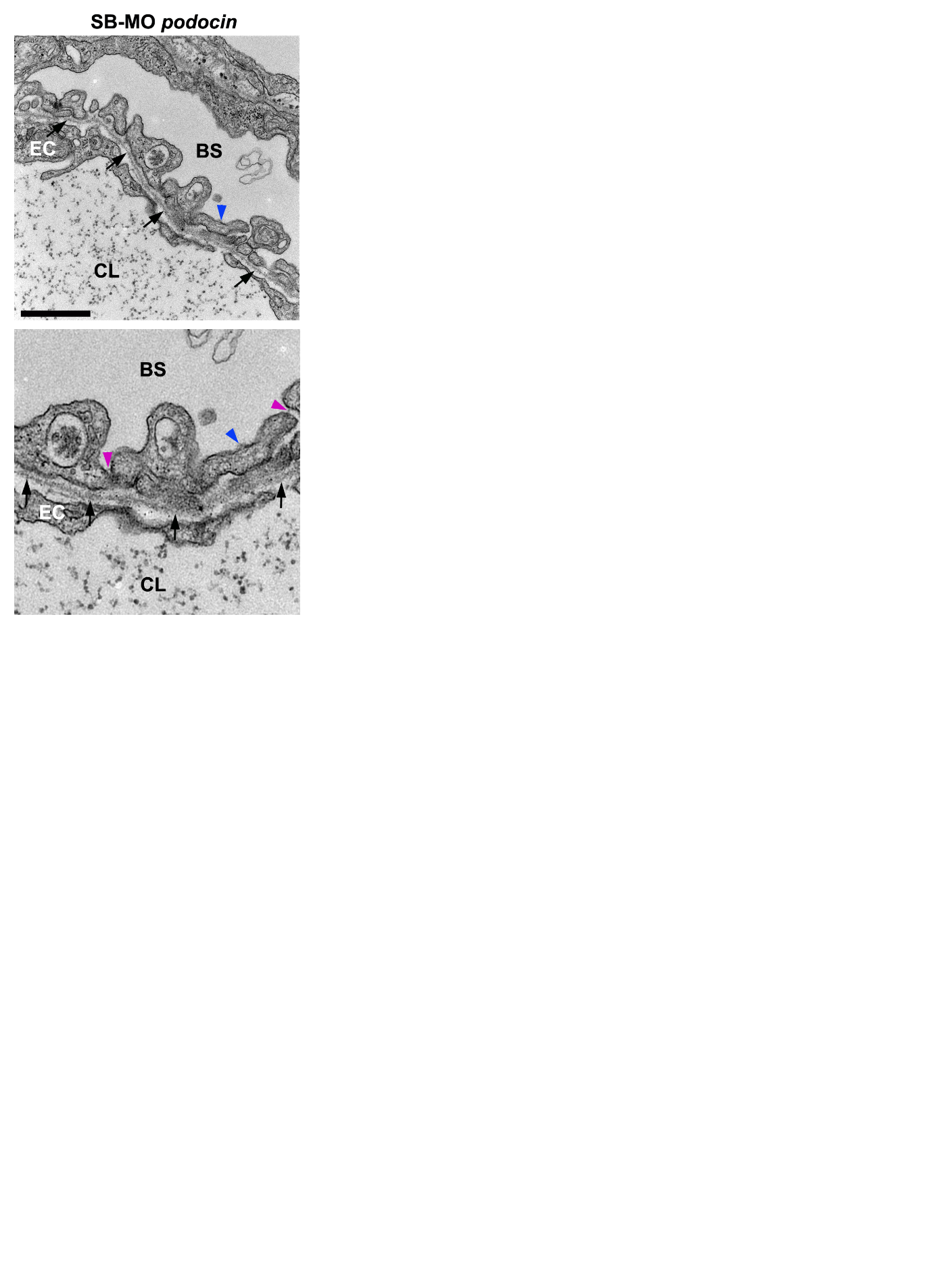


**Suppl. Fig. 2 Respective unprocessed gel images.**


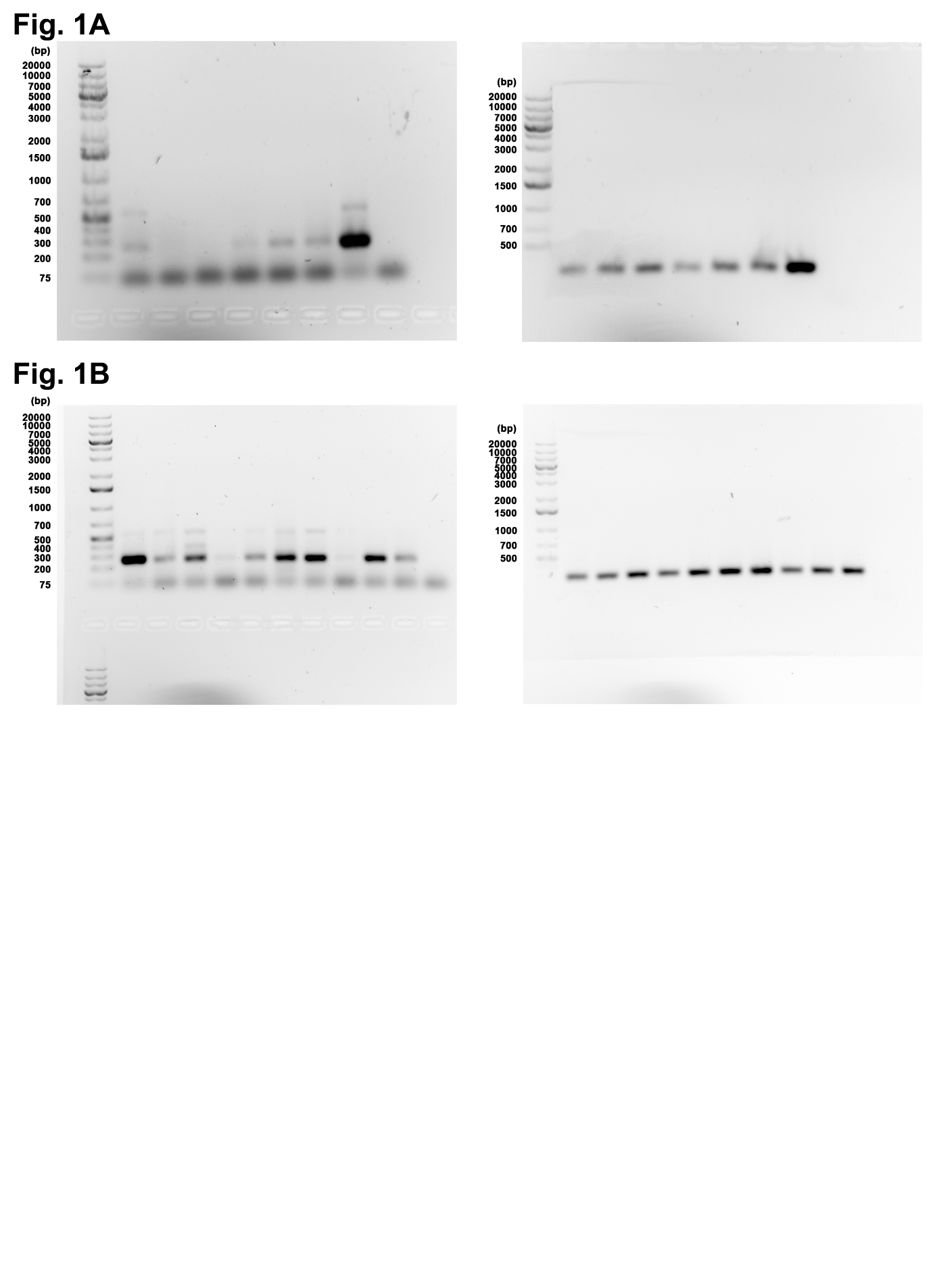


**Suppl. Fig. 3 Respective unprocessed blot and gel images.**


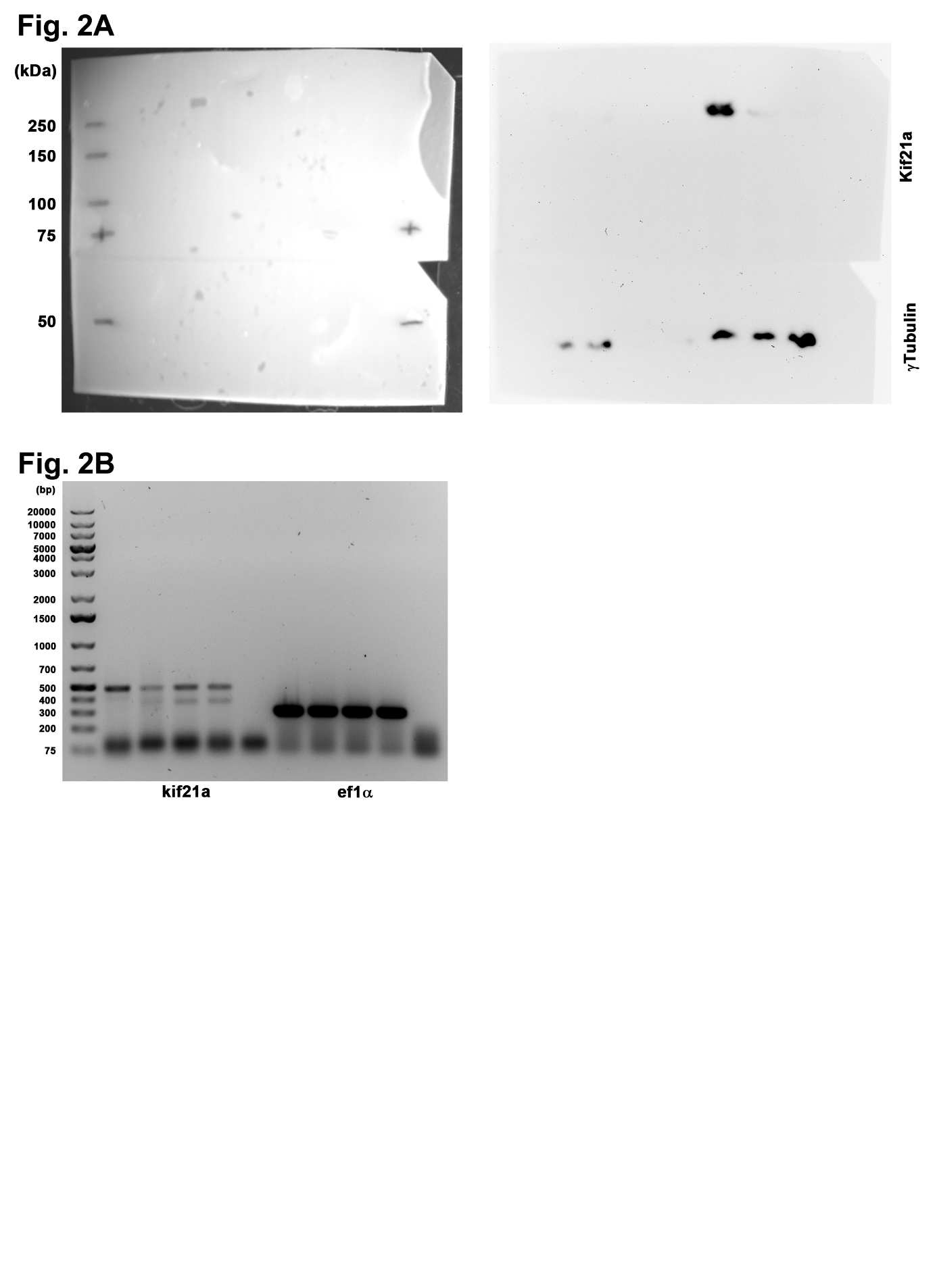


**Suppl. Fig. 4 Respective unprocessed SDS-PAGE images.**


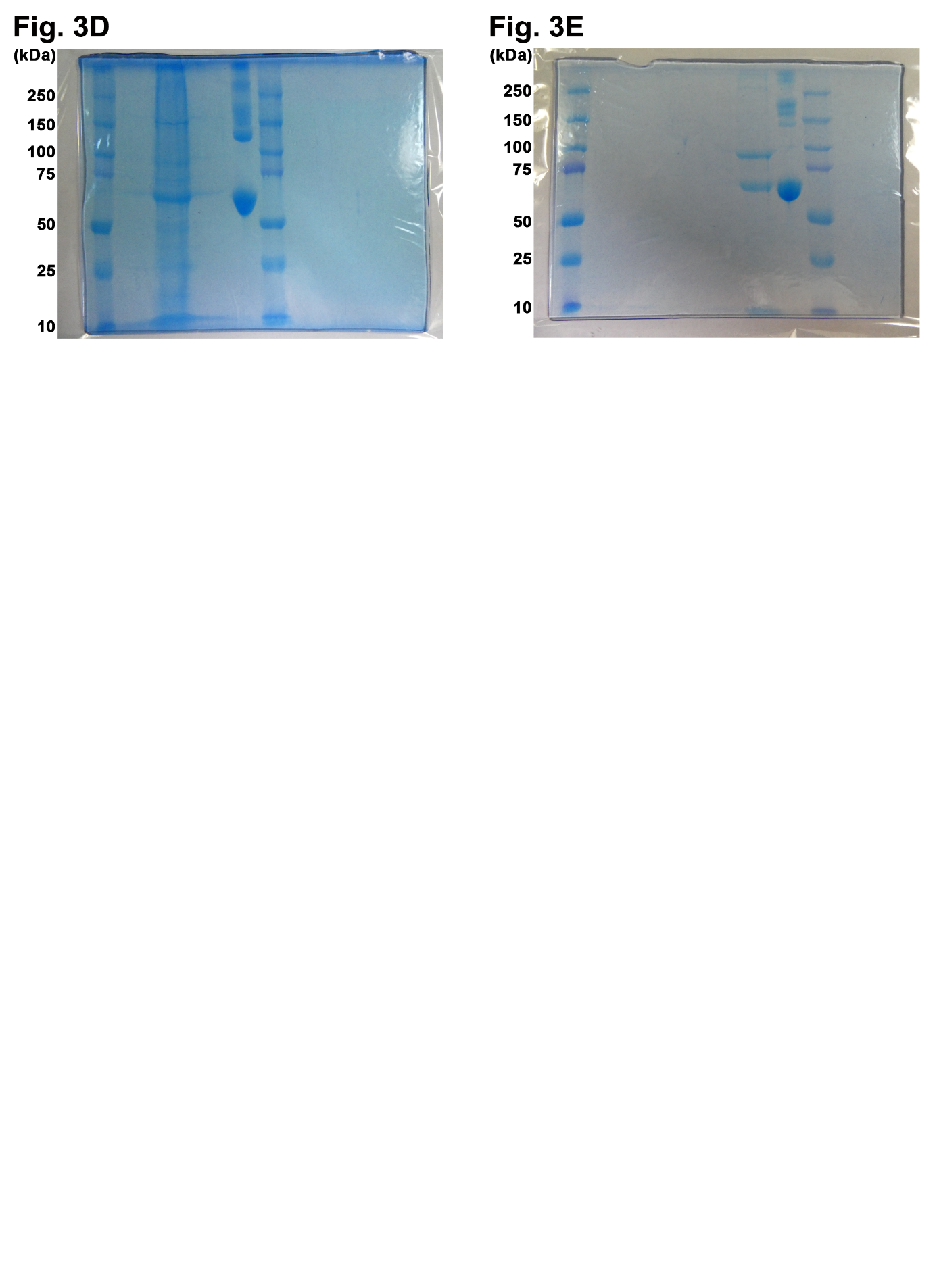


**Figure Legends**

**Suppl. Fig. 1 Podocin deficiency results in defective podocyte morphology.**

**Representative electron micrograph of glomerular region from 5dpf old zebrafish embryo injected with SB-MO *podocin* (1ng).** Blue and magenta arrowheads point to podocyte foot process effacements and slit diaphragms, respectively. Black arrows point to glomerular basement membrane. Bowman’s space (BS), capillary lumen (CL), fenestrated endothelial cell (EC)**. Scale bar represents 500nm.**
